# Effect of Hopcalite Modification on the Pore Textural and HCl Adsorption Properties of Activated Carbon Fibers

**DOI:** 10.3390/ma18214942

**Published:** 2025-10-29

**Authors:** Min Seong Han, Byong Chol Bai

**Affiliations:** Division of Energy Engineering, Daejin University, Pocheon 11159, Republic of Korea

**Keywords:** activated carbon fiber, adsorption HCl, hopcalite catalyst

## Abstract

Industrial air pollution, particularly acidic gases such as hydrogen chloride (HCl), poses serious environmental and health hazards. Here, hopcalite catalysts were introduced into activated carbon fibers via the impregnation process to enhance HCl capture. The Cu/Mn molar ratio was fixed at 1:1 while the Cu precursor loading was varied with the weight of Cu (Cu 0.04–0.1). Structural and surface modifications were examined using scanning electron microscope, energy-dispersive X-ray spectroscopy, X-ray photoelectron spectroscopy, inductively coupled plasma mass spectrometer, and Brunauer–Emmett–Teller analyses. Progressive CuMnOx deposition increased Cu and Mn contents up to 4 at.% and 3.7 at.%, respectively, but decreased the specific surface area from 1565.1 to 1342.7 m^2^/g owing to pore blocking. Fixed-bed breakthrough tests (50 ppm HCl, 1000 mL/min) showed that moderate catalyst addition (Cu 0.04) yielded the highest total removal (83.6%) and adsorption capacity (12,354.6 mg/g), benefiting from combined physical and catalytic chemisorption. Higher loadings (Cu 0.06–0.1) further reduced microporosity and led to lower removal efficiencies. These results demonstrate that an optimal CuMnOx level effectively promotes chemical adsorption without compromising the intrinsic microporous network of ACFs.

## 1. Introduction

In recent years, hydrogen chloride (HCl) emissions have been increasingly released into the atmosphere from various industrial activities, including direct HCl production, metal pickling, and the combustion or incineration of chloride-containing materials such as coal, plastics, and paper, as well as from manufacturing processes utilizing HCl as a reactant [1,2,3]. The emission of HCl gas poses significant risks to human health and the environment [4]. Inhalation can lead to eye irritation, skin discoloration, coughing, inflammation, suffocation, and ulceration of the upper respiratory tract [5]. Moreover, its strong corrosive nature can cause severe damage to metallic materials and limestone-based architectural structures [6]. To mitigate these environmental and health hazards, the development of efficient technologies for capturing and removing HCl gas has become increasingly important.

To capture and remove these harmful gases effectively, functional materials with large surface areas and high adsorption capacities are widely employed [7]. Representative examples include activated carbon, carbon nanotubes, and activated carbon fibers (ACFs), all of which are known to adsorb harmful pollutants effectively [8]. Among them, ACFs are fibrous carbonaceous materials that offer several advantages over other carbon adsorbents [9]. Micropore-rich ACFs generally exhibit strong physical adsorption capacity toward a wide range of gaseous pollutants, including volatile organic compounds (VOCs) [10]. Moreover, their well-developed microporous structure enables interaction with active sites without additional diffusion resistance from large pores, which typically governs the adsorption rate of granular adsorbents [11]. ACFs can be produced from a variety of precursors, including phenolic resins, polyacrylonitrile (PAN), viscose rayon, pitch, polyamide fibers, and other polymer fibers [12].

Adsorption can be classified into two types: physical adsorption and chemical adsorption [13]. In physical adsorption, gas molecules approach the pores of the adsorbent and are retained by van der Waals forces, whereas in chemical adsorption they are bound through chemical bonds formed between the adsorbate and the adsorbent [14,15]. The efficiency of physical adsorption primarily depends on pore structure and surface area, whereas chemical adsorption is governed by surface functional groups, atomic coordination, and electron density [16]. As a result, ACFs, with their abundance of well-developed micropores smaller than 2 nm, are particularly advantageous for physical adsorption [17]. However, the adsorption performance of adsorbents is not determined solely by their physical structure but is also strongly affected by surface chemistry [18]. Because of limited surface functional groups, ACFs are intrinsically nonpolar and thus exhibit restricted chemisorption and selectivity toward VOCs [19].

In order to improve the adsorption effect and selectivity, it is often necessary to adjust the pore structure of ACF or modify its surface characteristics [20]. Various surface modification strategies such as acid treatment, catalyst introduction, and plasma activation have been investigated to enhance the adsorption performance of ACFs [21]. Among them, catalysts act as agents that increase the rate of chemical reactions and reduce the activation energy of the reaction [22]. However, the introduction of excessive catalysts can form pore blockages, preventing reactants in various states from entering the pores [23]. Precious metals, nonmetals, and metal oxides are widely used as catalysts [24]. Among these catalysts, Hopcalite (CuMnOx), a mixed oxide of copper (Cu) and manganese (Mn), has been extensively used for low-temperature CO oxidation due to its excellent redox activity [25,26].

To date, various methods have been applied to prepare CuMnOx catalysts, including co-precipitation, sol–gel, reduction, and pyrolysis [27]. Among these methods, co-precipitation is one of the simplest and most useful for the preparation of CuMnOx catalysts [28]. This approach can produce highly active CuMnOx catalysts. The structure of a CuMnOx catalyst also depends on the preparation method, Cu:Mn molar ratio, drying temperature, and calcination conditions [29]. Copper-related oxygen species are highly active in CuMnOx catalysts [30]. The increase in catalytic activity is attributed to improved catalyst surface area, pore volume, and lattice oxygen mobility [31]. The lattice oxygen mobility of copper and manganese species increases catalyst reactivity [32]. Cu oxide alone is weakly active for CO oxidation, but when combined with Mn oxide in appropriate proportions, highly active catalyst systems can be generated [33].

Beyond CO oxidation, Cu-Mn oxides such as CuMnOx can also react with HCl through redox cycles involving Cu^2+^/Cu^+^ and Mn^4+^/Mn^3+^, leading to a chloride conversion process [34]. This chloride conversion represents irreversible chemical reactions that fundamentally alter the catalyst structure and oxidation state [35]. For example, MnO_2_ reacts with HCl and releases Cl at the same time as it is reduced to Mn^2+^ state, and CuO reacts with HCl to convert to CuCl_2_ [36,37]. However, few studies have systematically investigated how the incorporation of redox-active catalysts such as CuMnOx affects the adsorption-reaction behavior of acidic gases like HCl on carbon-based adsorbents. Therefore, it is necessary to understand how HCl gas interact through physical adsorption, chemical adsorption by catalysts, and chloride conversion processes in the ACF where CuMnOx catalysts are introduced.

In this study, a CuMnOx catalyst, a mixed oxide of copper and manganese, was introduced into ACFs by an impregnation method, and the resulting changes in pore structure and surface characteristics under different catalyst-loading conditions were examined. Subsequently, HCl gas, a representative acidic pollutant, was used to evaluate how pore structure and surface modification influence adsorption performance. This work aims not only to identify the optimal CuMnOx loading for maximum HCl removal but also to clarify the interplay between physical and chemical adsorption in CuMnOx-modified ACFs. The findings provide fundamental insight into designing high-efficiency carbon-based adsorbents for controlling acidic gases in industrial and environmental applications.

## 2. Materials and Methods

### 2.1. Materials

Activated carbon fiber (ACF, Jiangsu Sutong Carbon Fiber Co., Ltd., STF-1500, Nantong, China) with a specific surface area of 1500 m^2^/g are used as catalyst supports, Cu(NO_3_)_2_∙3H_2_O (Copper(II) nitrate trihydrate, 99.0%, Daejung, Siheung, Republic of Korea), Mn(NO_3_)_2_∙6H_2_O (Manganese(II) nitrate hexahydrate, 97.0%, Junsei, Tokyo, Japan) and Al(NO_3_)_3_∙9H_2_O (Aluminum nitrate nonahydrate, 98.0%, Daejung, Siheung, Republic of Korea) were used to produce catalysts for CuMnOx. HCl gas (50 ppm; N_2_ gas was used as a balance; Rigas, Daejeon, Republic of Korea) was used for the adsorption performance. Finally, N_2_ gas was used to remove the remaining HCl gas.

### 2.2. Preparation of Hopcalite-Modified ACFs

The CuMnOx catalysts were synthesized by impregnation method. In 50 mL of distilled water, Cu(NO_3_)_2_∙3H_2_O, Mn(NO_3_)_2_∙6H_2_O and Al(NO_3_)_3_∙9H_2_O are put in beaker at a fixed rate and stirred sufficiently until dissolved. Here, Al(NO_3_)_3_·9H_2_O was introduced as a precursor for alumina, which acted as a support to promote uniform dispersion and interfacial stability of the CuMnOx species on the ACF surface. The molar ratio of (Cu:Mn) to the CuMnOx catalyst was (1:1), and the overall weight ratio of Cu(NO_3_)_2_∙3H_2_O:Mn(NO_3_)_2_∙6H_2_O:Al(NO_3_)_3_∙9H_2_O was 1:1.10:0.98. Immerse 10 × 10 cm ACF in a mixed solution and dry overnight at 80 °C oven. The obtained ACF is raised to 450 °C at a temperature rise of 10 °C/min in a nitrogen atmosphere carbonizing furnace and fired for 5 h. The sample name is attributed to the amount of Cu(NO_3_)_2_∙3H_2_O and is indicated as Raw, Cu 0.04, Cu 0.06, Cu 0.08 and Cu 0.1.

### 2.3. HCl Gas Adsorption

**Figure 1:**
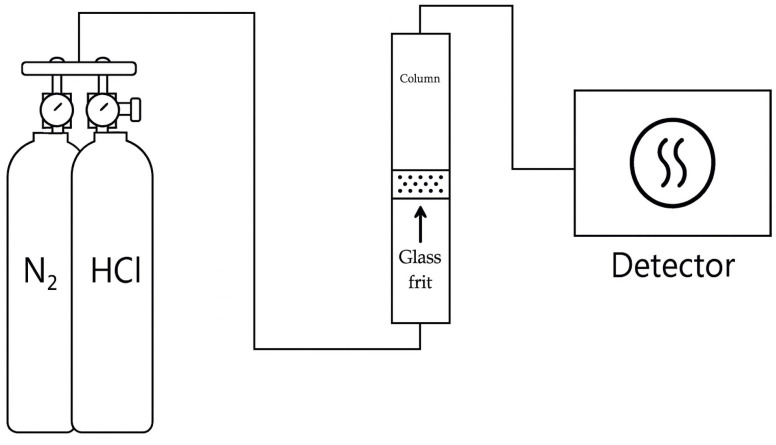
Schematic diagram of experimental set up.

Before loading the ACF sample, the glass column and gas lines were purged with N_2_ to remove residual gases and impurities. The ACF was then packed into a glass column (inner diameter 1.6 cm) to form a fixed bed of 2.5 cm height. A test stream containing 50 ppm HCl was introduced at a flow rate of 1000 mL/min. The outlet HCl gas concentration was continuously measured every second using the IR-based HCl gas analyzer. A schematic diagram of the experimental set up is shown in Figure 1.

### 2.4. Characterization of Hopcalite-Modified ACFs

Scanning electron microscopy (SEM; VEGA3, Tescan, Kohoutovice, Czech Republic) was used to observe that the CuMnOx catalyst was introduced into the ACF. To examine the elements distribution of the ACF where CuMnOx was introduced, an analysis was conducted using Energy-dispersive X-ray spectroscopy system (EDS, AZtecEnergy ver. 5.1 with X-Max SDD, Oxford Instruments, Abingdon, UK) equipped within the SEM. Inductively couples plasma mass spectrometer (ICP-MS; Agilent 7900, Agilent Technologies, Santa Clara, CA, USA) was used to analyze the manganism and copper elemental composition of the CuMnOx catalyst. After the catalyst was introduced, the specific surface area and micropore volume of the ACF were determined from low-temperature nitrogen adsorption–desorption isotherms using the Brunauer–Emmett–Teller (BET; Tristar 3020, Micromeritics, Norcross, GA, USA) method. After the introduction of CuMnOx catalysts into the ACFs, x-ray photoelectron spectroscopy (XPS; Nexsa, Thermo Fisher Scientific Brno, Waltham, MA, USA) analysis was conducted to evaluate and analyze the chemical composition of the surface. Finally, to evaluate the adsorption performance of the ACFs, the adsorption characteristics were analyzed by calculating the time and concentration values using an HCl gas detector (FIX800, Wandi, Korea, Gunpo-si, Republic of Korea) with 50 ppm HCl gas.

## 3. Results and Discussion

### 3.1. Morphology of Hopcalite-Modified ACFs

**Figure 2:**
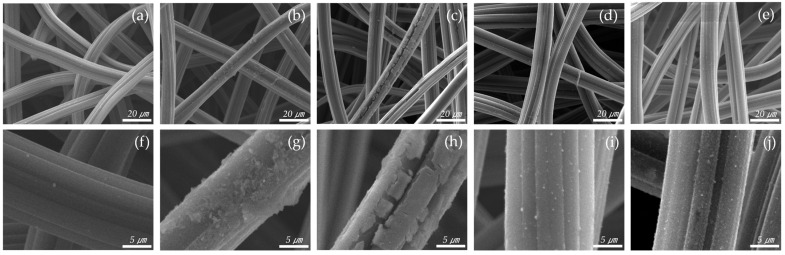
SEM scanning images of hopcalite-modified ACFs (**a**,**f**) RAW, (**b**,**g**) Cu 0.04, (**c**,**h**) Cu 0.06, (**d**,**i**) Cu 0.08, (**e**,**j**) Cu 0.1.

After the catalyst was introduced, the surface structure of the ACF was observed by SEM, as shown in Figure 2. The thickness of the fiber strands was about 15 µm. As the amount of catalyst increased, it could be seen that the catalyst gradually formed on the fibers. The Cu 0.04 sample shows that catalysts were deposited to form particles on the ACF surface. The Cu 0.06 sample allows visual confirmation that most of the surface was coated with catalyst, but the coating was not complete, resulting in cracks. The Cu 0.08 and Cu 0.1 samples indicate that the ACF was completely coated with the catalyst. Subsequent analyses were conducted to determine the amount of catalyst in each sample.

### 3.2. Elemental Analysis of Hopcalite-Modified ACFs

**Figure 3:**
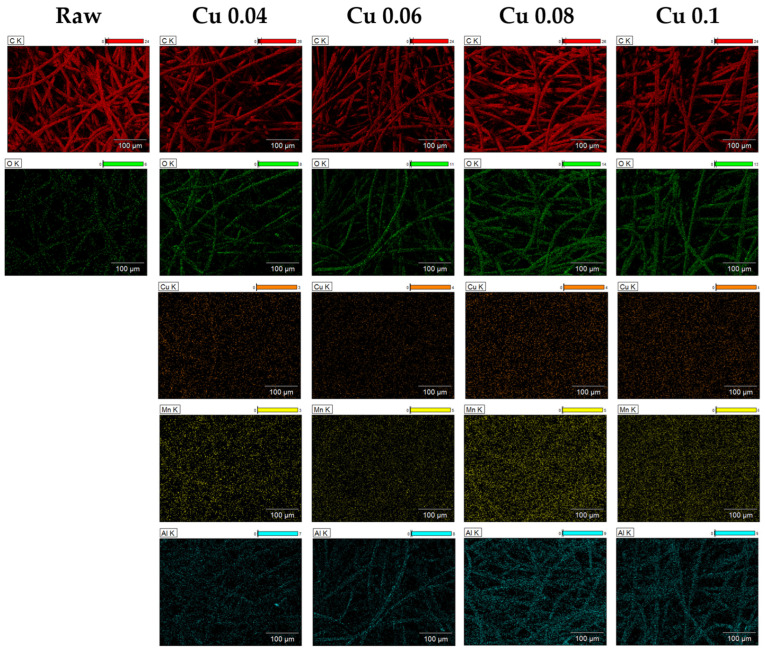
EDS images of hopcalite-modified ACFs.

EDS and XPS analyses were performed to examine the elemental composition of the ACF surface. As shown in Table 1, the carbon content of ACF without catalysts was 95.31%, consisting mostly of carbon. After the catalyst was introduced, the contents of Cu, Mn, Al, and O increased, confirming that the catalyst was successfully incorporated into the ACF. The atomic ratio of carbon decreased accordingly, and these changes are illustrated by the elemental mapping in Figure 3. The mapping shows that, as the catalyst content increases, the colors representing the other elements become more distinct. C, O, and Al elements were mainly distributed around the fiber surface, while Cu and Mn were spread across the entire area.

**Figure 4:**
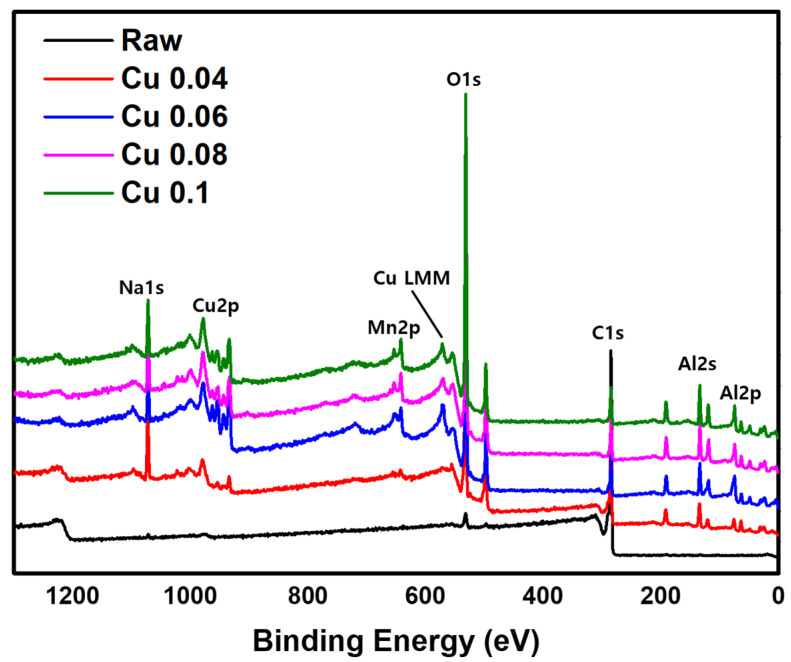
XPS image of hopcalite-modified ACFs.

The XPS analysis results are shown in Figure 4. A weak C1s peak at a binding energy of 284.8 eV and a strong O1s peak at 530.0 eV are observed [38,39]. When a catalyst is introduced, the O1s peak becomes stronger. The catalyst components Cu2p and Mn2p appear at binding energies of 934.3 eV, 954.3 eV, and 643.6 eV, respectively, and the support Al2p shows a peak at 73.9 eV [40,41,42]. In addition, the Al2s and Na1s peaks are observed at 118.9 eV and 1072.0 eV, respectively [43,44]. The auger LMM peaks of Cu^+^, and Cu^0^ are located at 569.9 eV and 567.9 eV, respectively [45]. Na1s peak is identified as being formed during the production of ACF. Together with the EDS data, these XPS results confirm that the catalyst was successfully introduced into the ACF. However, because XPS is a surface-sensitive technique, it is difficult to quantify the total catalyst content. Therefore, ICP-MS analysis was conducted for quantitative evaluation.

The ICP-MS data from the quantitative analysis of the catalyst are shown in Table 2. Similarly to the EDS and XPS results, ICP-MS analysis shows that the elemental contents increase with higher catalyst loading. In the Raw sample, Cu and Mn are present only in trace amounts. In contrast, the Cu 0.1 sample with the highest catalyst content contains 32,293.23 ppm of Mn and 46,057.65 ppm of Cu. The quantitative analysis was limited to Cu and Mn because these two elements serve as the active components directly responsible for the catalytic performance. Therefore, determining the Cu and Mn loadings was sufficient to verify the successful incorporation of the precursors and to evaluate the effect of catalyst loading on the overall composition. BET analysis was then performed to determine how the catalyst affects the pores and specific surface area of the ACF.

### 3.3. Specific Surface Area of the Hopcalite-Modified ACFs

**Figure 5:**
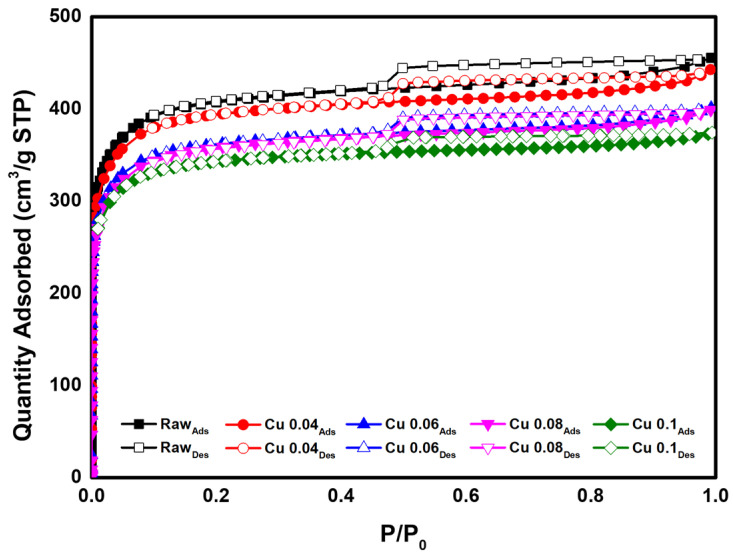
Adsorption isotherm of hopcalite-modified ACFs.

**Figure 6:**
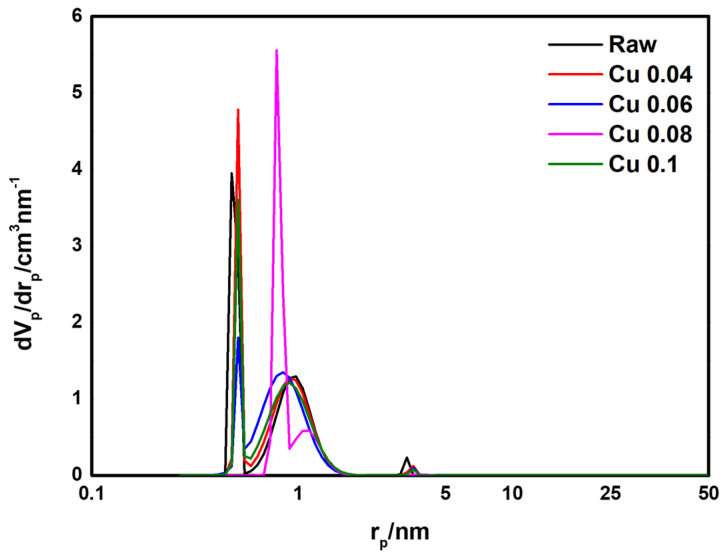
Pore size distribution of hopcalite-modified ACFs.

BET analysis was performed to determine the effect of the catalyst on the specific surface area and pore volume of the ACF. The results are presented in Table 3 and Figure 5. According to the adsorption isotherm analysis in Figure 5, all graphs correspond to Type I in the IUPAC classification, indicating that most pores are micropores of 2 nm or less [46]. As the amount of added catalyst increased, the BET surface area decreased from 1565.1 m^2^/g to 1342.7 m^2^/g. This decrease is attributed to catalyst deposition and the resulting pore blockage in the ACF. The total pore volume decreased from 0.70 cm^3^/g to 0.58 cm^3^/g. Except for the Cu 0.1 sample, the micropore percentage remained above 95%. Figure 6 shows the pore size distribution of ACFs according to catalyst loading. As shown in Table 3, with microporosity above 90%, most pores are confirmed to be micropores of 2 nm or less. Subsequently, HCl gas adsorption characterization was performed to evaluate how catalyst loading, specific surface area, and pore structure influence adsorption.

### 3.4. HCl Gas Adsorption Properties

Breakthrough curves (*C_t_*/*C*_0_ vs. time) were drawn to analyze the adsorption of HCl in the upflow fixed-bed column; the data were evaluated using the following equations (Effluent volume: *V_eff_*) [47]:

(1) $$V_{eff}=Qt_{total}$$

where *t_total_* represents the total operation time (min) and *Q* represents the flow rate (mL/min). The total uptake capacity (*q_total_*) is given by Equation (2) [48]:

(2) $$q_{total}=\frac{Q}{1000}\int_{t=0}^{t=t_{total}}C_{0}dt$$

where *C_ad_* is the concentration of HCl gas adsorbed in the ACF. The total amount of HCl flowing into the column (*m_total_*, mg) is defined as follows [49]:

(3) $$m_{total}=\frac{C_{0}V_{eff}}{1000}$$

The total HCl removal (*R_total_*, %) was calculated as follows [49]:

(4) $$R_{total}=\frac{q_{total}}{m_{total}}\times 100$$

The mass transfer zone (*MTZ*), *Z_m_*, is defined as follows [50]:

(5) $$Z_{m}=Z\left(1-\frac{t_{0.05}}{t_{0.95}}\right)$$

where *Z_m_* is the length of the *MTZ* (cm), *Z* is the bed height (cm), *t*_0.05_ is the time at the breakthrough (min), and *t*_0.95_ is the time at saturation (min). The column usage of the fixed-bed column adsorption device (*ƒ*) is defined as follows [50]:

(6) $$ƒ=\left(1-\frac{0.5\times MTZ}{bed\;height}\right)\times 100$$

**Figure 7:**
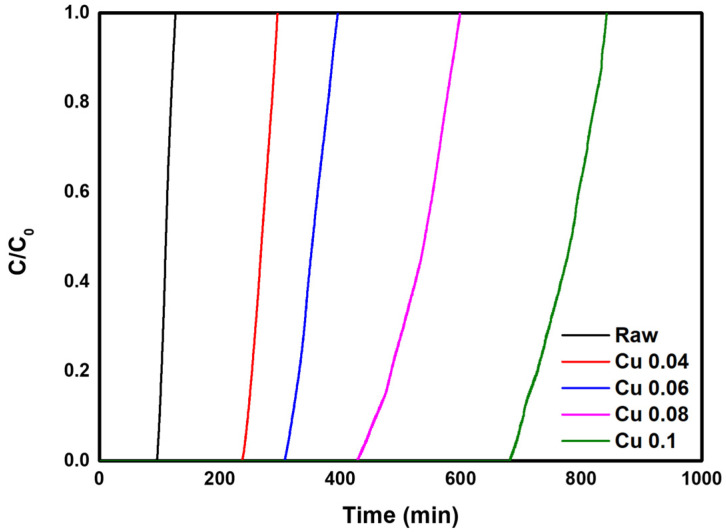
HCl gas breakthrough curve of the hopcalite-modified ACFs.

In this study, experiments were conducted to evaluate the HCl adsorption characteristics of five samples in which ACFs were modified with different catalyst loadings. Data on HCl adsorption and breakthrough curves are shown in Table 4 and Figure 7. From the calculated values in Table 4, it was confirmed that the adsorption amount initially increased as the catalyst content increased. The Cu 0.04 sample showed the highest total removal at 83.63%, followed in order by Raw > Cu 0.06 > Cu 0.08 > Cu 0.1. These results indicate that adsorption in unmodified ACFs occurs mainly by physical adsorption. In the Cu 0.04 sample, which had the lowest catalyst content, the catalyst slightly reduced the specific surface area (Table 3) but without significant impact. Nevertheless, total removal increased compared with the decrease in specific surface area. When the change in surface area is small, the catalyst appears to have little effect on the physical adsorption of ACFs while promoting chemical adsorption and thus increasing total removal. In contrast, in the Cu 0.06, Cu 0.08, and Cu 0.1 samples, the specific surface area decreased markedly because of the higher catalyst content, reducing the physical adsorption capacity and lowering total removal. In addition, based on the length of the *MTZ*, the bed utilization factor showed high efficiency of nearly 90% for most samples. Overall, these results confirm that both catalyst loading and the specific surface area and pore structure significantly influence gas adsorption characteristics. An appropriate catalyst amount can enhance chemical adsorption without impairing physical adsorption, whereas excessive catalyst loading interferes with physical adsorption and reduces overall adsorption. This suggests that an optimal catalyst content must be carefully controlled.

The mechanism of HCl removal over CuMnOx-modified ACFs can be explained by the combined effects of physical and chemical adsorption. In the initial stage, HCl molecules are physically adsorbed onto the microporous surface of ACF through van der Waals interactions, which are mainly governed by its high surface area and micropore volume. Subsequently, the CuMnOx catalyst promotes chemical adsorption via redox reactions involving Cu^2+^/Cu^+^ and Mn^4+^/Mn^3+^ species. The representative reactions can be expressed as:

MnO_2_ + 4HCl → MnCl_2_ + 2H_2_O + Cl_2_

CuO + 2HCl → CuCl_2_ + H_2_O

These results indicate that HCl is not only physically adsorbed on the ACF surface but also chemically converted into chloride compounds through oxidation–reduction processes. Therefore, the overall HCl removal mechanism is considered to be a synergistic process, in which microporous physical adsorption and catalytic redox reactions occur simultaneously to enhance total removal efficiency.

## 4. Conclusions

This study evaluated the HCl adsorption characteristics of activated carbon fibers with a specific surface area of 1500 m^2^/g after introducing hopcalite catalysts by an impregnation method under four different conditions. SEM analysis confirmed that the more catalysts were introduced, the more thoroughly they covered the activated carbon fibers. Subsequent surface elemental analyses showed that as the catalyst content increased, the amounts of Cu, Mn, Al, and O also increased. In addition, ICP-MS provided quantitative analysis and, consistent with the EDS and XPS results, confirmed that higher catalyst loading led to higher concentrations of Cu and Mn.

BET analysis revealed that the specific surface area decreased as the catalyst content increased. For the Cu 0.1 sample with the highest catalyst loading, the specific surface area was reduced to 1342.7 m^2^/g. All samples, however, maintained more than 90% micropores with pore sizes of 2 nm or less.

Finally, HCl gas adsorption tests demonstrated how the catalyst affected the adsorption characteristics of the activated carbon fibers. The adsorption capacity decreased as the specific surface area declined, while the Cu 0.04 sample achieved the highest total removal of 83.63%. Samples with greater catalyst loadings than Cu 0.04 sample showed lower adsorption capacity than unmodified fibers. Bed utilization was also confirmed to be nearly 90% efficient. These findings show that adsorption capacity depends on both the presence of the catalyst and the specific surface area.

## Figures and Tables

**Table 1:** Surface characteristics of hopcalite-modified ACFs.

	Atomic (%)				
	Raw	Cu 0.04	Cu 0.06	Cu 0.08	Cu 0.1
C (%)	95.31	82.34	76.93	74.56	70.18
O (%)	4.69	13.45	16.80	18.24	20.25
Cu (%)	0	2.22	2.65	2.90	3.98
Mn (%)	0	1.19	2.29	2.72	3.66
Al (%)	0	0.80	1.33	1.58	1.93

**Table 2:** Quantitative analysis of hopcalite-modified ACFs by ICP-MS.

	Raw	Cu 0.04	Cu 0.06	Cu 0.08	Cu 0.1
Cu (ppm)	163.13	10,318.37	25,849.31	30,582.99	46,057.65
Mn (ppm)	155.84	5038.85	9697.20	19,828.92	32,293.23

**Table 3:** BET surface properties of hopcalite-modified ACFs.

Sample	S_BET_ (m^2^/g)	Total Pore Volume (cm^3^/g)	Micropore Volume (cm^3^/g)	Microporosity (%)
Raw	1565.1	0.70	0.67	95.71
Cu 0.04	1507.2	0.68	0.65	95.59
Cu 0.06	1386.3	0.62	0.60	96.77
Cu 0.08	1367.8	0.61	0.59	96.72
Cu 0.1	1342.7	0.58	0.53	91.38

**Table 4:** HCl gas adsorption characteristic of the hopcalite-modified ACFs.

Sample Name	Superficial Velocity	Initial Concentration	Bed Height	Total Time	Breakthrough Time	Saturation Time	Effluent Volume	Total Amount Removed	Total Removal	Adsorption Capacity	Length of MTZ	Bed Utilization
	*Q*	*C*_0_	*Z*	*t_total_*	*t*_0.05_	*t*_0.95_	*V_eff_*	*m_total_*	*R_total_*	*q_total_*	*L_MTZ_*	*f*
	(mL/min)	(mg/L)	(cm)	(min)	(min)	(min)	(cc)	(mg)	(%)	(mg/g)	(cm)	(%)
Raw	1000	50	2.5	125.8	97.6	124.1	125,816	6290.8	74.70	4699.3	0.53	89.34
Cu 0.04	1000	50	2.5	295.5	242.3	293.3	295,450	14,772.5	83.63	12,354.6	0.44	91.29
Cu 0.06	1000	50	2.5	395.7	314.2	391.1	395,660	19,783.0	70.28	13,903.6	0.49	90.17
Cu 0.08	1000	50	2.5	598.6	443.6	593.2	598,600	29,930.0	65.11	19,488.3	0.63	87.39
Cu 0.1	1000	50	2.5	842.2	693.2	838.3	842,200	42,110.0	62.84	26,460.3	0.43	91.35

## Data Availability

The original contributions presented in this study are included in the article. Further inquiries can be directed to the corresponding author.
